# A Case of Spinal Epidural Abscess Presenting with Horner Syndrome

**DOI:** 10.7759/cureus.14541

**Published:** 2021-04-18

**Authors:** Wala O Sati, Mahmoud Haddad, Shahzad Anjum

**Affiliations:** 1 Emergency Medicine, Hamad General Hospital, Doha, QAT; 2 Emergency Medicine, Hamad Medical Corporation, Doha, QAT

**Keywords:** spinal epidural abscess, horner syndrome, neurological deficit

## Abstract

A spinal epidural abscess (SEA) is an uncommon disease, but it is associated with significant morbidity. SEA can be promoted by multiple risk factors. Moreover, the diagnosis of SEA usually requires the presence of a classic triad of back pain, fever, and neurological deficit, hence, the difficulty in making the diagnosis if presented otherwise. Horner syndrome (HS) is an uncommon presentation in association with SEA. Even though nonsurgical versus surgical management of SEA is still controversial, the literature review indicates a preference for surgical decompression as a treatment for SEA presenting with neurological compromise, followed by long-term antimicrobial therapy. The rapidity of making the diagnosis and the initiation of appropriate treatment determine the outcome.

We present a case of a 23-year-old male with no past medical history. The patient arrived at the Hamad General Hospital emergency department (ED) with severe upper back pain radiating to his left shoulder, which progressed to numbness and weakness of the left upper limb and spastic paraplegia. A left HS was revealed in a further neurological examination. However, the diagnosis of a spinal epidural abscess (SEA) was made after a left posterolateral epidural abscess extending from C5/6-T2/3 with associated cord compression and edema was revealed on an MRI scan. The patient then underwent a left C7, T1 hemilaminectomy and received antibiotics followed by admission to the rehabilitation unit. *Staph. aureus *was reported in culture microbiology results. Unfortunately, motor power recovery after the surgery was not significant.

Although it is difficult to diagnose SEA, it is crucial to suspect it in the presence of a neurological deficit regardless of the presence or absence of predisposing factors. Nevertheless, HS is not a relatively common finding in association with SEA. In this case report, we have a young patient with SEA who presented with left HS, upper back pain, and progressive neurological deficit in the absence of identifiable risk factors.

## Introduction

A spinal epidural abscess (SEA) is a suppurative infection of the central nervous system. Patients typically present with midline back pain, fever, and neurologic deficits, but many cases present atypically [1]. A case series of 36 SEA cases demonstrates that SEAs are commonly found in the thoracolumbar spine, which has large epidural spaces and contains more infection-prone adipose tissue [2].

The incidence of SEA has been increasing over the past decade. Rigamonti et al. documented approximately 12.5 cases per 10,000 admissions at a large referral center [3]. This increased incidence can be linked to the aging of the general population and the growing number of patients with predisposing factors such as intravenous drug abuse and human immunodeficiency virus (HIV) [1].

SEA is difficult to diagnose if clinical suspicion is not high. Radiological imaging is the key to diagnosis, imaging modes of choice are CT and MRI with contrast [1]. In 2002, a study reported that the initial accurate diagnosis rate was as low as 26% [4]. However, the most common presenting complaint in patients with SEA is back pain followed by fever [5].

Although the definitive management of SEA is generally provided by a spine surgeon, the early suspicion and establishment of the diagnosis and early initiation of treatment by an emergency physician are crucial factors in the outcome. Surgery within 36-72 hours from the onset of neurological deficits is considered an early surgery. The majority of studies supported early surgery to prevent permanent morbidity and mortality rather than providing medical treatment alone, which is associated with 41%-42.5% treatment failure rates [6]. In the absence of a neurological deficit, medical management is an alternative to surgery when the risk of neurological complications is low based on the abscess location and morphology, patient immune status, and the causative organism [5]. Therefore, it is essential for all physicians to recognize the features of SEA, be able to initiate an appropriate laboratory and radiological workup, and have an understanding of the basic management principles [1].

## Case presentation

A 23-year-old male arrived at our emergency department with a complaint of upper back pain radiating to the left shoulder along with progressive left upper limb pain over a course of 10 days, which began after he pushed his bed. His symptoms advanced to develop numbness involving the inner side of his left upper limb, followed by gradual weakness of bilateral lower limbs over the next five days.

The patient’s symptoms worsened as he developed drooping of his left eyelid and, lastly, he lost his ability to walk. He also reported decreased sensations of a full bladder and while passing urine. However, the patient had no other complaints, including fever.

Upon examination at the emergency department, the patient was afebrile with normal physiological parameters for his age. The neurological examination showed partial left eye ptosis with a 1 mm pupil size, while the size of the right pupil was 3 mm. Although anhidrosis was not tested, the diagnosis of left Horner syndrome (HS) was considered. In comparison to the normal power of the right upper limb, the power over the left upper limb was impaired with grade 3/5, and bilateral lower limb spastic paraparesis and hyperreflexia were present in the absence of clonus and Hoffman sign. Furthermore, the sensory system was also affected at the C8 level on the left side, and crude touch and pinprick sensation were diminished bilaterally from the T3 level and downwards. Digital rectal examination revealed a significant decrease in anal tone and perianal sensation. Also, urinary bladder distention was confirmed by point-of-care ultrasound (POCUS). Laboratory results reported elevated CRP level at 132 (normal value <5) and a normal white blood cell (WBC) of 10.4 x10^3/uL with 8.8 x10^3/uL (88%) neutrophils.

**Figure 1:**
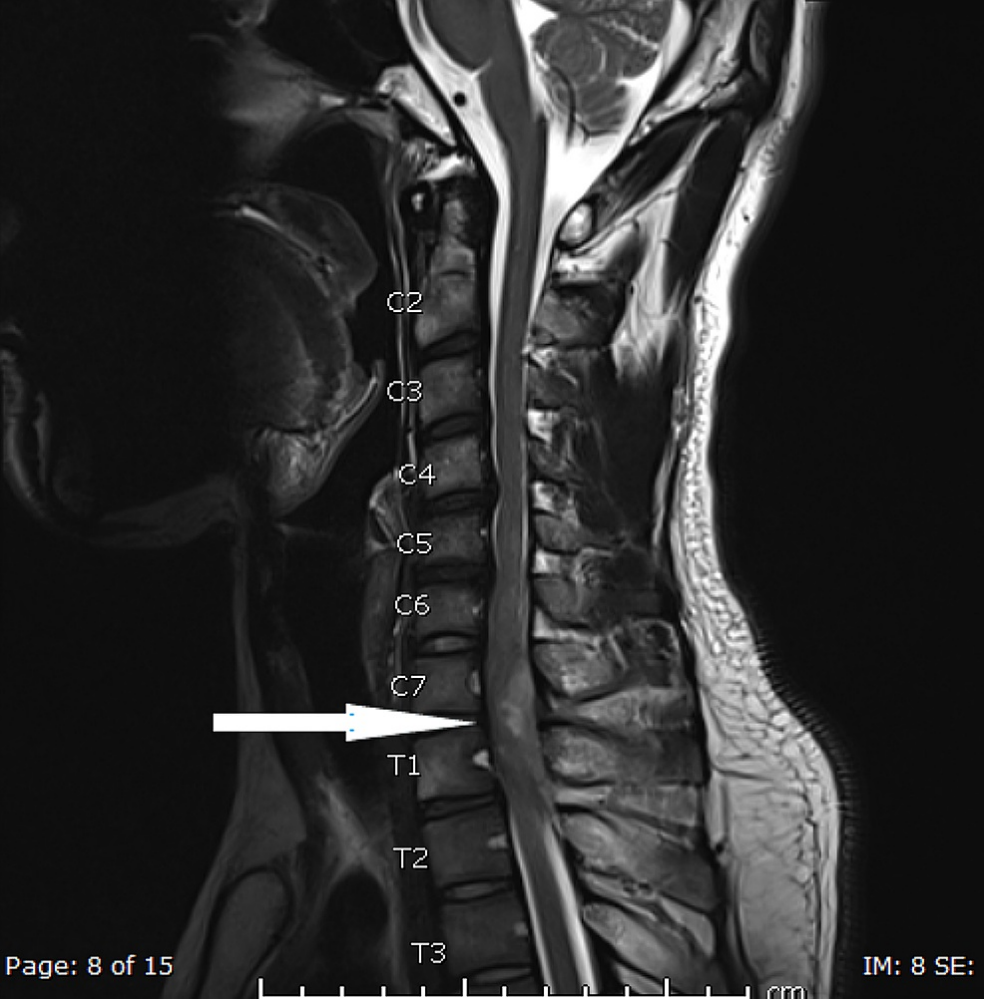
T2 Sagittal view showing extramedullary collection identified extrinsic to the spinal cord extending from C5/6 down to T2/3, placed posterolaterally on the left side and measuring 57 x 18 x 11 mm

**Figure 2:**
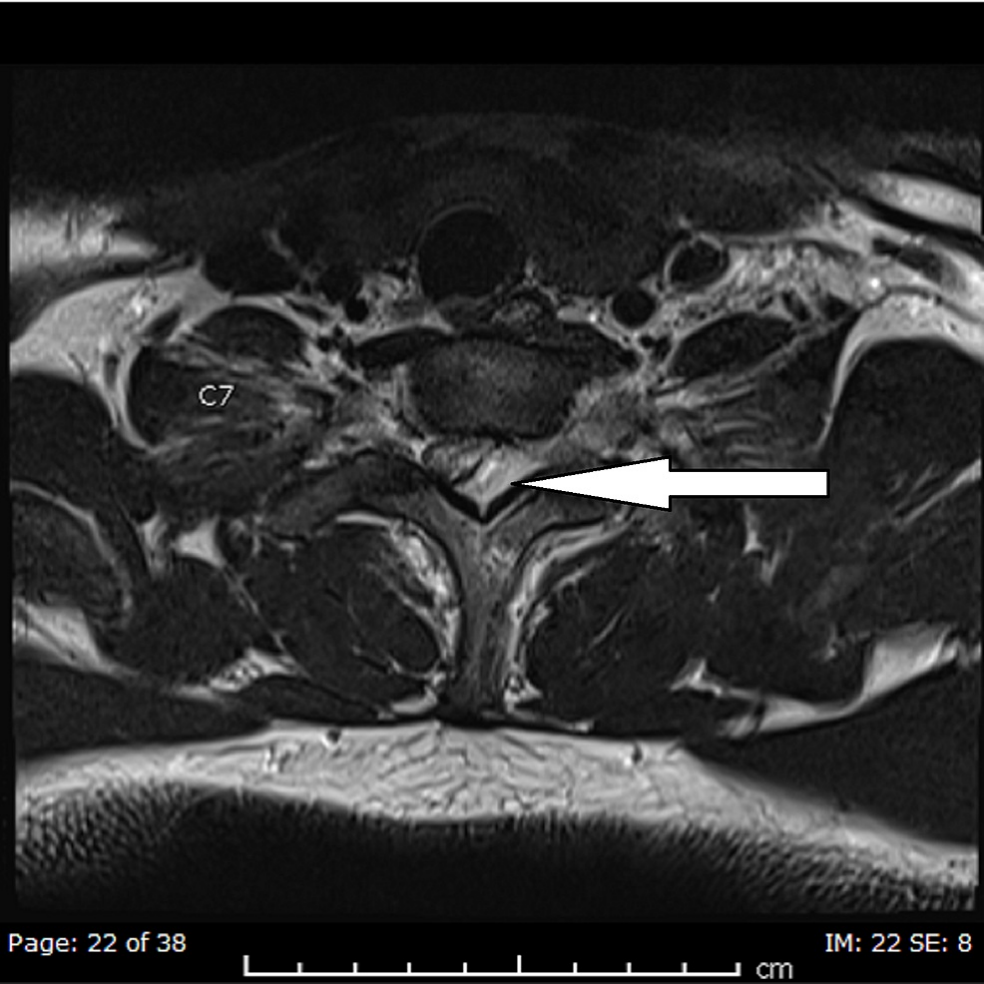
T2 axial image

**Figure 3:**
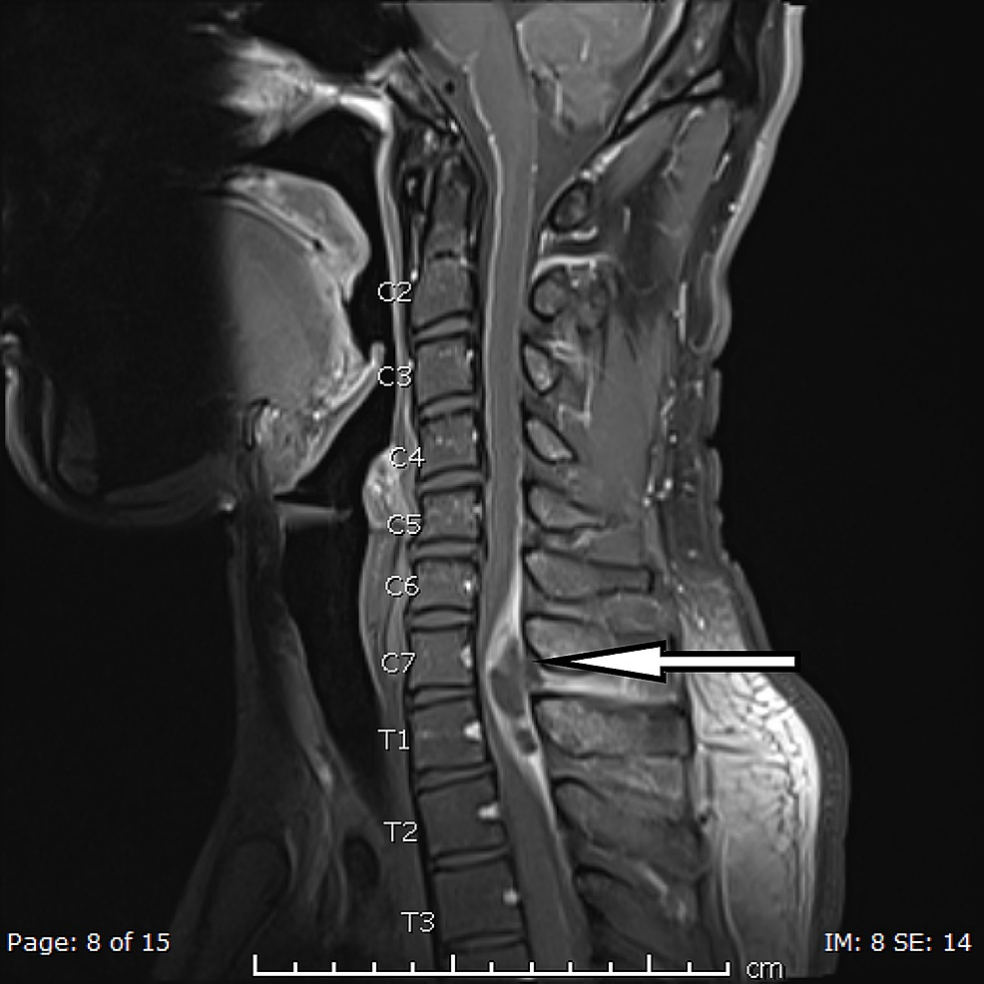
T1 fat saturation post-contrast sagittal view representing enhancement is predominantly peripheral with central necrosis There is an anterolateral displacement of the spinal cord to the right side with resultant cord compression and associated cord T2 signal hyperintensity.

**Figure 4:**
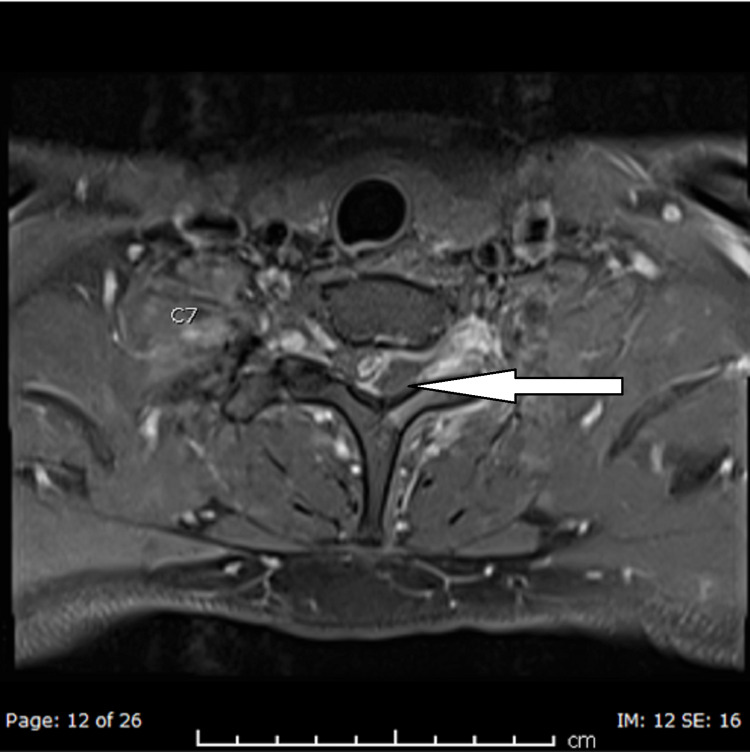
T1 post-contrast axial view

A cervical spine X-ray to rule out bony pathology or abnormal vertebral alignment was conducted and reported normal, whereas an MRI with contrast study performed for the neurological deficit showed a left extrinsic posterolateral spinal cord collection, measuring 57 x 18 x 11 mm, was extending from C5/6 down to T2/3. Spinal cord displacement with secondary cord compression was noted. Also, there was adjacent soft-tissue enhancement, indicating associated inflammatory changes extending to the perivertebral and interspinous region of C6-T2. Associated reactive osteomyelitis was identified in the C6-T1 spinous processes, and moderate impingement on the left C6 nerve root was noted by a left lateral C5/6 disc protrusion. However, the alignment of the spine was satisfactory. MRI films are shown in Figures 1-4.

The patient was admitted to the neurosurgery department and received IV cefazolin. He underwent a left-sided C7 and T1 hemilaminectomy to relieve pressure on spinal nerves. The operative findings included pus collection with granulation tissue adherent to the dura mater. The pus was drained and cultures were sent for analysis. Afterward, culture results of the pus displayed moderate *Staph. aureus* growth that was sensitive to clindamycin, erythromycin, trimethoprim/sulfamethoxazole (TMP/SXM), tetracycline, cefazolin, and cloxacillin. Postoperatively, there was a significant improvement in touch sensation over the lower limbs due to the release of compression. In contrast, the improvement in motor power after the surgery was negligible.

The patient was admitted to a rehabilitation facility nine days after the surgery. In rehab, the patient underwent a multidisciplinary rehabilitation program with physical and occupational therapy and a bladder and bowel training program. On discharge, he had persistent grade +1 lower limb spasticity and hypertonicity, ambulating on a wheelchair, and on four-hourly intermittent urinary bladder catheterizations for urinary bladder incontinence. The muscle tone and power of the upper limb were recovered. Unfortunately, there was no sufficient documentation about the HS after admission except for the persistent ptosis.

## Discussion

Our aim from presenting this case report is to emphasize the importance of suspecting SEA in patients presenting to the ED atypically with neurological deficits irrespective of the presence of a risk factor. SEA is a rare but potentially devastating condition that requires a high index of suspicion. Early diagnosis and treatment correlate to a greater outcome. Delayed diagnosis or insufficient treatment may lead to severe and/or permanent disabilities [7].

SEA incidence has a bimodal distribution pattern peaking below 20 years and between 50 and 70 years of age. The male-to-female ratio ranges between 2:1 and 5:1 [7]. Comorbidities that increase the risk of developing SEA include intravenous drug abuse, diabetes mellitus, liver disease and other infections, cardiovascular or renal disease and dialysis, tobacco consumption, recent dental procedures, and immunosuppression [8].

Hematogenous spread is the most common route of spread, followed by skin infections in up to 25% of patients. Additionally, teeth, respiratory tract and middle ear, genitourinary tract, endocarditis, or phlebitis are considerable sources. Direct spread from an adjacent tissue of bony vertebral column, intervertebral disc, or paravertebral soft tissue due to previous spinal surgeries may occur, or due to an epidural or spinal puncture procedure [5]. In the younger age group, drug abuse is the most common cause [9].

Blunt trauma and cutaneous infections may result in developing bacterial SEA, where blunt trauma was reported preceding SEA symptoms in 15%-35% of cases [10]. It is presumed that trauma may result in epidural hematoma formation, which subsequently becomes infected [11].

The most common causative microorganism, *Staphylococcus aureus*, accounts for about 70% of cases. This is followed by *Streptococcus *species, which account for approximately 7% of cases. Even though *gram-negative bacilli *are less common, they are often isolated from intravenous drug abusers. On the other hand, *Mycobacterium tuberculosis*, fungal species, and parasitic organisms are rare causes of SEA, particularly without associated vertebral osteomyelitis. Sterile cultures are seen in up to 40% of patients [5]. A tuberculous abscess is usually the sole manifestation of latent tuberculosis reactivation [10]. In this case, trauma was suspected to be the possible predisposing factor since the patient's symptoms started with back pain after pushing a heavy object, followed by rapid progression of neurological deficit.

SEA has variable clinical manifestations. Back pain is the most frequent complaint presenting in 70%-90% of patients. Fever was reported in 60%-70% of individuals. A neurological deficit was found in almost one-third of patients with SEA [5]. When the clinical picture is masked by sepsis, presenting features may include jaundice, fever, confusion, and disseminated intravascular coagulation (DIC) [11]. On physical examination, localized tenderness on the spine is often found, whereas the severity of neurological deficit fluctuates between monoradiculopathy and a complete cord lesion. However, the progression of SEA occurs in four phases, starting with spinal pain with or without nerve root pain and spinal tenderness, followed by motor weakness and sensory loss, impaired sphincter control, and, lastly, complete paralysis [5].

An abnormal plain X-ray is found in only 30%-70% of cases. CT is beneficial, as it may reveal bony destruction with extradural lesions when MRI is unavailable or inappropriate. However, the investigation of choice for SEA is contrast-enhanced MRI because it offers excellent visualization of the abscess, vertebra, disc space, and/or paraspinal infection foci [12].

Whenever SEA is suspected, instant treatment initiation is required. Conservative management can be considered in (1) patients unfit for surgeries, (2) extreme length of SEA (holospinal), (3) patients suffering from minor or no neurological deficits, or (4) delayed presentation with complete paralysis for more than 72 hours. However, the gold standard treatment has been proven to be the combination of surgical decompression with systemic antibiotics. Intravenous antibiotic administration should be applied for two to four weeks, followed by oral antibiotics. The duration and route of antibiotic administration are still debatable, with a suggested total treatment period of six to 12 weeks [7].

Successful treatment results with antibiotics alone have been reported in a few studies of selected patients; however, the majority of these patients had no to mild neurological deficits and smaller abscesses [12]. In case of prolonged paraparesis - more than four days - the outcome is poor regardless of the infectious agent [9].

Surgical treatment is indicated in the presence of (1) spinal cord compression, (2) significant spinal deformity, (3) poor response to nonoperative treatment, or (4) difficulty in obtaining a bacteriological diagnosis or close biopsy. Surgical treatment consists of debridement of the infected foci, thecal sac decompression, with or without spinal stabilization. The most important procedure is radical debridement of the affected tissues with decompression of the thecal sac, but it is accompanied by the risk of spinal instability [13].

HS is primarily a clinical diagnosis, which represents a group of signs that occurs when interruption of the sympathetic pathway occurs [14]. HS is caused by the interruption of the oculosympathetic pathway between the hypothalamus and orbit. The classical triad of HS includes ipsilateral miosis, ptosis, and facial anhidrosis [9]. HS can result from any level interruption at the sympathetic fibers supplying the eye and face [15]. In a case series of 450 patients with HS, 270 (60%) had an identifiable cause [16]. Some common causes of HS according to the location of injury/lesion are listed in Table 1.

**Table 1:** Anatomical location, pathological processes, and clinical manifestation in Horner syndrome lesion localization [14]

Neuronal order	Location	Pathology	Associated clinical signs
First-order (central syndrome)	Posterior hypothalamus Brainstem	Pituitary tumors Stroke—Wallenberg pontine hemorrhage/ Demyelination	Vertigo Altered facial sensation Contralateral CN IV palsy Crossed Radicular signs motor/sensory signs
	C-spine Intermediolateral grey substance C8-T2	Arnold-Chiari Cervical spondylosis Syringomyelia Neck trauma	
Second-order (intermediate)	C8–T2 ventral nerve roots	Cervical rib	Neck/arm pain and weakness Brachial plexus injury
	Apex of the lung	Tumors, Pancoast tumor, mesothelioma	Signs of lung disease
	Mediastinum	Cardiothoracic procedure Aortic aneurysm or dissection	Vocal cord paralysis, Anhidrosis of face and neck
	Cervical sympathetic chain	Subclavian artery aneurysm, Thyroid tumor, Post-neck dissection	
Third-order (postganglionic)	Superior cervical ganglion at C2-3	Jugular venous ectasia	Facial pain, Stroke, Ocular ischemia
	Carotid artery	Carotid dissection, Aneurysm, Arteritis	
	Cavernous sinus	Base of skull tumors	Abducens palsy
	Inflammatory mass orbit	Herpes zoster	

The underlying pathology of HS will be apparent in over 80% of patients at the time of the first neuro-ophthalmic consultation based on the history or possible location of the lesion [14]. Furthermore, a painful HS is quite a common ocular presentation, which has been reported in up to 58% of cases with internal carotid artery (ICA) dissection [17]. Isolated HS is frequently benign or from a previously identified cause. However, carotid dissection and neoplasm are considerable underlying causes [14].

A review study conducted on 25 cases with intramedullary spinal cord abscesses was able to detect a single case presenting with HS in addition to the motor and sensory neurological deficits, back pain, bladder dysfunction, and fever in the presence of risk factors (diabetes mellitus and lumbar puncture procedure), which was reported in 1982 [18]. In contrast to our case report, our patient had no obvious recognizable risk factor for developing SEA and the presumed underlying cause for developing HS was related to spinal nerve root compression by the abscess collection, cord compression, and adjacent tissue edema.

## Conclusions

Although SEA is a rare disease, early recognition with the initiation of treatment and timely evacuation reduces neurological morbidity and potential mortality. The data on SEA presenting with isolated HS or in association with other neurological deficits is currently inadequate. The index of suspicion of SEA is typically low in the absence of the SEA classic triad and risk factors, hence the struggle to establish the diagnosis and initiate the appropriate therapy. Subsequently, this may result in permanent neurological deficit and mortality. Moreover, most patients with SEA have one or more predisposing factors. This case report highlights that SEA can present atypically in a healthy young individual with no single predisposing factor, and initial unawareness of the condition may lead to delayed diagnosis, hence the poor outcome.
